# Development of spasticity with age in a total population of children with cerebral palsy

**DOI:** 10.1186/1471-2474-9-150

**Published:** 2008-11-06

**Authors:** Gunnar Hägglund, Philippe Wagner

**Affiliations:** 1 Department of Orthopaedics, Lund University Hospital, S-221 85 Lund, Sweden; 2 Swedish National Competence Centre for Musculoskeletal Disorders, Lund University Hospital, S 221 85 Lund, Sweden

## Abstract

**Background:**

The development of spasticity with age in children with cerebral palsy (CP) has, to our knowledge, not been studied before. In 1994, a register and a health care program for children with CP in southern Sweden were initiated. In the programme the child's muscle tone according to the modified Ashworth scale is measured twice a year until six years of age, then once a year. We have used this data to analyse the development of spasticity with age in a total population of children with cerebral palsy.

**Methods:**

All measurements of muscle tone in the gastrocnemius-soleus muscle in all children with CP from 0 to 15 years during the period 1995–2006 were analysed. The CP subtypes were classified according to the Surveillance of Cerebral Palsy in Europe network system. Using these criteria, the study was based on 6218 examinations in 547 children. For the statistical analysis the Ashworth scale was dichotomized. The levels 0–1 were gathered in one category and levels 2–4 in the other. The pattern of development with age was evaluated using piecewise logistic regression in combination with Akaike's An Information Criterion.

**Results:**

In the total sample the degree of muscle tone increased up to 4 years of age. After 4 years of age the muscle tone decreased each year up to 12 years of age. A similar development was seen when excluding the children operated with selective dorsal rhizotomy, intrathecal baclofen pump or tendo Achilles lengthening. At 4 years of age about 47% of the children had spasticity in their gastro-soleus muscle graded as Ashworth 2–4. After 12 years of age 23% of the children had that level of spasticity. The CP subtypes spastic bilateral and spastic unilateral CP showed the same pattern as the total sample. Children with dyskinetic type of CP showed an increasing muscle tone up to age 6, followed by a decreasing pattern up to age 15.

**Conclusion:**

In children with CP, the muscle tone as measured with the Ashworth scale increases up to 4 years of age and then decreases up to 12 years of age. The same tendency is seen in all spastic subtypes. The findings may have implications both for clinical judgement and for research studies on spasticity treatment.

## Background

The spastic subtypes are the most common manifestations of cerebral palsy (CP) [1]. Spasticity is defined as "a velocity-dependent increase in tonic stretch reflexes (muscle tone) with exaggerated tendon jerks, resulting from hyperexcitability of the stretch reflex, as one component of the upper motor neurone syndrome" [2].

It is well known that children with spastic types of CP often have a normal or low muscle tone in the newborn period [3]. During the first years of life the muscle tone increases, and by age 4 it should be possible to diagnose the spasticity in all children with spastic CP [4]. However, the further development of spasticity with age has, to our knowledge, not been studied before.

In 1994, a cerebral palsy register and a health care programme were initiated for children with CP in southern Sweden [5,6]. The child's local physiotherapist fills in a recording form twice a year until six years of age, then once a year. The recording form includes measurement of muscle tone according to the modified Ashworth scale [7] (Table 1).

**Table 1:** The modified Ashworth scale

**Score**	**Description of the muscle tone**
0	Normal tone, no increase in tone
1	Slight increase in tone – a catch and release at the end of the range of motion
1+	Slight increase in tone – catch, followed by minimal resistance in remainder of range
2	More marked increase in tone through most of range
3	Considerable increase in tone, passive movement difficult
4	Affected parts rigid in flexion or extension

We have used this data to analyse the development of spasticity with age in a total population of children with cerebral palsy.

## Methods

The CPUP register includes all children with CP born after 1 January 1990 living in the counties of Skåne and Blekinge in southern Sweden, which have a total population of about 1.3 million. The number of children with CP in the area corresponds to a prevalence of 2.4 per 1000 living births [8,9]. Since 2005, CPUP has been a National Health Care Quality Register approved by the National Board of Health and Welfare in Sweden.

The programme includes a continuing standardized follow-up of gross and fine motor function, clinical findings and treatment. The child's local physiotherapists examine the child and fill in the recording form twice a year until six years of age, then once a year. There are 13 child habilitation units in the area, with about 80 physiotherapists involved in the CPUP-programme. The recording form includes the CP subtype, as well as measurement of muscle tone according to the modified Ashworth scale [7] (Table 1). The measurements are performed in stated and standardized positions described in a manual connected to the recording form.

In the present study all measurements of muscle tone in the gastrocnemius-soleus muscle in all children with CP from 0 to 15 years of age during the period 1995 – 2006 were analysed. The measurements were done with the child lying prone with the hip and knee extended. Only children with definite diagnosis of CP were included. The CP subtypes were classified according to the Surveillance of Cerebral Palsy in Europe network (SCPE) [10]. In children with unilateral spastic CP, only the spastic side was used in the analysis. In all other cases both sides were used.

**Figure 1:**
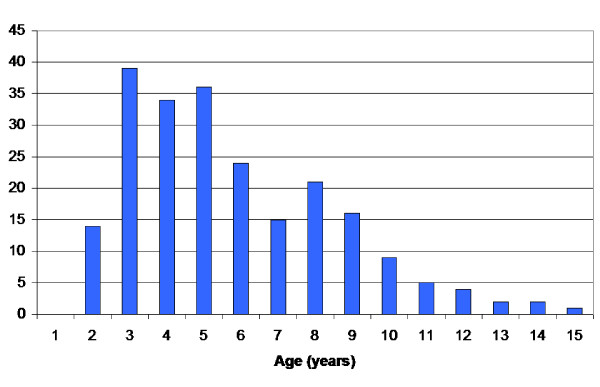
Number of treatments with BxA related to age at time of treatment in the total sample of children with CP.

Using these criteria, the study was based on 6218 examinations in 547 children. The distribution of CP subtypes and measurements are presented in Tables 2 and 3. The children treated with selective dorsal rhizotomy (SDR), intrathecal baclofen pump (ITB), tendo Achilles or gastrocnemius lengthening (TAL) are known (Table 4). The results are presented with these children both included and excluded. During the period 104 children were treated with botulinumtoxin A (BxA) of the gastrocnemius-soleus muscle on 262 occasions. The distribution of age at time of treatment is presented in Figure 1. In most cases the measurement of muscle tone was done more than three months after BxA treatment. The children treated with BxA are included in the study.

**Table 2:** Distribution of children and examinations in relation to CP subtypes based on the Surveillance of Cerebral Palsy in Europe (SCPE).

**CP subtype**	**Children**	**Examinations**
Spastic		
unilateral	186	1269
bilateral	266	3521
Dyskinetic	72	882
Ataxic	53	546
Total	547	6218

**Table 3:** Distribution of examinations in relation to age (years) and CP subtypes.

**Age**	Spastic unilateral	Spastic bilateral	Dyskinetic	Ataxic	Total
1	37	58	30	6	131
2	84	242	75	20	421
3	130	369	102	40	641
4	152	469	105	48	774
5	187	482	92	71	832
6	139	359	92	54	644
7	88	284	60	44	476
8	97	250	50	45	442
9	88	243	50	38	419
10	71	198	54	34	357
11	60	172	42	32	306
12	49	147	44	36	276
13	46	122	38	30	236
14	27	72	26	28	153
15	14	54	22	20	110
Total	1269	3521	882	546	6218

**Table 4:** Number of children (examinations) treated with selective dorsal rhizotomy (SDR), intrathecal baclofen pump (ITP) or tendo Achilles lengthening (TAL).

**CP subtype**	**SDR**	**ITB**	**TAL**	**Total**
Spastic				
unilateral			24 (211)	24(211)
bilateral	32 (594)	10 (194)	27 (441)	66 (1165)
Dyskinetic		6 (84)	4 (64)	9 (132)
Ataxic			1 (20)	1 (20)
**Total**	32 (594)	16 (278)	56 (736)	100 (1528)

Three children have been treated with both TAL and SDR and one child with both TAL and ITB.

In the statistical analysis the Ashworth scale was dichotomized. Levels 0, 1 and 1+ were gathered in one category and levels 2, 3 and 4 in the other. The analysis then focused on estimating the age effects on the risk of sustaining an Ashworth level of 2 or above. It was implemented using the logistic regression algorithms in STATA 9.0 [11]. Consideration of the correlation structure imposed by the inclusion of data on both legs for most children was given through the use of robust estimates.

In order to allow for a potential change in the direction of the development of spasticity with age, linear splines were used in the logistic regression analysis. The linear splines construction allows for a change in the estimated increase or decrease of risk with age, at a pre-specified age. Different placement and combinations of turning points in the development with age were evaluated using Akaike's An Information Criterion (AIC) [12].

Intuitively one can think of this process as determining the age development pattern most likely to have generated the data, while correcting for the number of parameters used to describe it. The correction is needed since an increase in parameters tends to enhance the pattern fit to data without reflecting any valuable information. Therefore the number generated by the AIC can be thought of as corresponding to the probability that the given development pattern generated the data, penalized with respect to its number of parameters.

Furthermore, since a perceived change in the risk of sustaining an Ashworth level of 2 or above, could be a consequence of late inclusion and early dropout of different birth cohorts, having been exposed to different methods of treatment, consideration was also given the difference between the actual longitudinal age effects of interest, and cross-sectional birth cohort effects. This possibility was evaluated by jointly estimating age and birth cohort effects in the logistic regression analysis. Results from the analysis can be considered to reflect the average development of spasticity with age taken over each of the birth cohorts.

The estimates produced in the analysis can be interpreted as relative risks. For instance, when a result states that the odds ratio is 1.5 from 0 to 3 years of age and 0.80 from 3 to 9 years of age, the relative risk interpretation of the estimates finds that the risk increases by 50% each year up to the age of 3. It then diminishes by 20% each year from then on up until the age of 9.

## Results

The analysis of the development of spasticity using Akaike's AIC is presented in Table 5, and the relative risk estimates are presented in Tables 6 and 7.

**Table 5:** Analysis of the development of spasticity with age using AIC [12].

**Population**	**AIC**
**Total population**	

No turning point	1.2952
Turning point at 4 years	1.2921
Turning point at 4 and 12 years	1.2902

Total population – children operated* excluded	

No turning point	1.2893
Turning point at 6 years	1.2838
Turning point at 6 and 12 years	1.2822

**Spastic unilateral CP**	

No turning point	1.1901
Turning point at 4 years	1.1795
Turning point at 4 and 11 years	1.1698

Spastic unilateral CP-children operated* excluded	

No turning point	1.2099
Turning point at 4 years	1.1948
Turning point at 7 and 7 years	1.1918

**Spastic bilateral CP**	

No turning point	1.3338
Turning point at 3 years	1.1317
Turning point at 3 and 12 years	1.3305

Spastic bilateral CP – children operated* excluded	

No turning point	1.3508
Turning point at 7 years	1.3469
Turning point at 7 and 12 years	1.3449

**Dyskinetic CP**	

No turning point	1.3605
Turning point at 6 years	1.3448

Dyskinetic CP – children operated* excluded	

No turning point	1.3301
Turning point at 6 years	1.2928

In each subgroup the AIC value for an age development pattern without turning point, an age development pattern with turning points and age at turning points that yielded the lowest AIC value are presented.

* Children operated with SDR, ITB and/or TAL excluded

**Table 6:** Relative risk estimates i.e. the risk of sustaining a level of spasticity greater than or equal to 2 as compared to the year before, in different subgroups and age periods.

**Population**	**Age period and relative risk estimates**		
**Total population**	**< 4 yrs**	**4–12 yrs**	**> 12 yrs**

	1.15(1.06–1.26)*	0.87(0.85–0.89)*	1.08(0.97–1.19)

**Total population – op excluded**^1^	**< 6 yrs**	**6–12 yrs**	**> 12 yrs**

	1.08(1.02–1.13)*	0.84(0.80–0.87)*	1.06(0.94–1.20)

**Spastic unilateral CP**	**< 4 yrs**	**4–11 yrs**	**> 11 yrs**

	1.44(1.18–1.76)*	0.78(0.73–0.84)*	1.24(1.03–1.50)*

**Spastic unilateral CP – op excluded**^1^	**< 4 yrs**	**4–7 yrs**	**> 7 yrs**

	1.63(1.27–2.08)*	0.73(0.62–0.85)*	0.94(0.86–1.04)

**Spastic bilateral CP**	**< 3 yrs**	**3–12 yrs**	**> 12 yrs**

	1.33(1.07–1.64)*	0.88(0.86–0.91)*	1.06(0.93–1.21)

**Spastic bilateral CP – op excluded**^1^	**< 7 yrs**	**7–12 yrs**	**> 12 yrs**

	1.08(1.00–1.12)	0.83(0.77–0.89)*	1.09(0.92–1.27)

**Dyskinetic CP**	**< 6 yrs**	**> 6 yrs**	

	1.15(1.03–1.27)*	0.84(0.74–0.97)*	

**Dyskinetic CP – op excluded**^1^	**< 6 yrs**	**> 6 yrs**	

	1.23(1.09–1.39)*	0.76(0.70–0.83)*	

In brackets 95% confidence interval.

* p < 0.05 for the two-sided test of the relative risk being equal to 1.

^1 ^excluded operations; SDR, ITB, TAL

**Table 7:** Relative risk estimates i.e. the odds of sustaining a level of spasticity greater than or equal to 2 as compared to the year before, in different subgroups and age periods.

**Population**	**Age period and relative risk estimates**		
**Total population**	**< 4 yrs**	**4–12 yrs**	**> 12 yrs**
	1.10(1.00–1.20)*	0.85(0.82–0.87)*	1.07(0.97–1.20)
**Total population – op excluded**^1^	**< 6 yrs**	**6–12 yrs**	**> 12 yrs**
	1.04(0.98–1.10)	0.84(0.80–0.88)*	1.10(0.96–1.25)
**Spastic unilateral CP**	**< 4 yrs**	**4–11 yrs**	**> 11 yrs**
	1.29(1.05–1.58)*	0.76(0.71–0.82)*	1.29(1.05–1.57)*
**Spastic unilateral CP – op excluded**^1^	**< 4 yrs**	**4–7 yrs**	**> 7 yrs**
	1.46(1.14–1.88)*	0.65(0.54–0.77)*	0.96(0.87–1.06)
**Spastic bilateral CP**	**< 3 yrs**	**3–12 yrs**	**> 12 yrs**
	1.24(1.00–1.55)*	0.86(0.83–0.86)*	1.08(0.94–1.024)
**Spastic bilateral CP – op excluded**^1^	**< 7 yrs**	**7–12 yrs**	**> 12 yrs**
	1.04(0.98–1.11)	0.83(0.77–0.90)*	1.13(0.95–1.33)
**Dyskinetic CP**	**< 6 yrs**	**> 6 yrs**	
	1.28(1.13–1.45)*	0.82(0.76–0.88)*	
**Dyskinetic CP – op excluded**^1^	**< 6 yrs**	**> 6 yrs**	
	1.39(1.21–1.60)*	0.80(0.73–0.88)*	

Estimates corrected for potential differences in risk of separate birth cohorts. In brackets 95% confidence interval.

* p < 0.05 for the two-sided test of the relative risk being equal to 1.

^1 ^excluded operations; SDR, ITB, TAL

**Figure 2:**
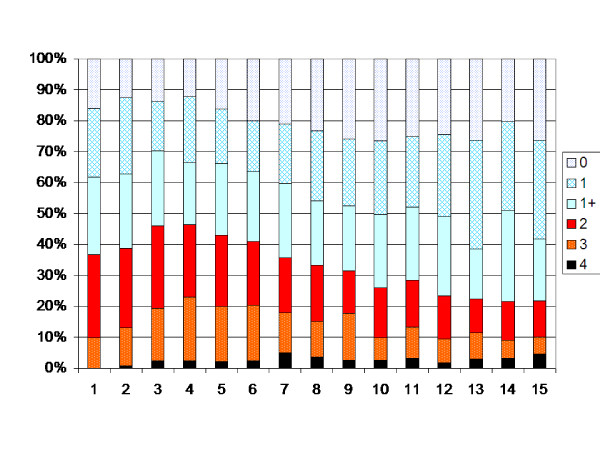
**Degree of spasticity in the gastrocnemius-soleus muscle according to the modified Ashworth scale related to age in the total sample of children with CP (547 children, 6218 measurements).** Number of measurements presented as a percentage of the total number of measurements in each age group.

**Figure 3:**
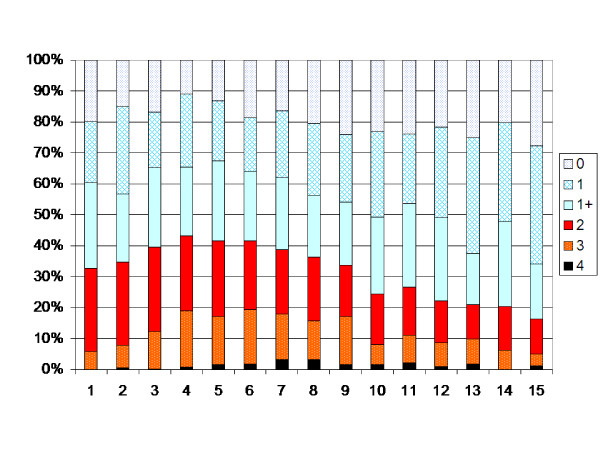
**Degree of spasticity in the total sample according to Figure 2.** Children treated with SDR, ITB or TAL excluded.

In the total population the degree of muscle tone increased up to 4 years of age (p < 0.05). After 4 years of age the muscle tone decreased each year up to 12 years of age (p < 0.05). After 12 years of age no significant change in development is seen (Figure 2). The analysis of the possible effect of different birth cohorts did not show any differences explaining the pattern of development (Table 7). When the children operated with SDR, ITB or TAL were excluded, the turning points were 6 and 12 years, i.e. the degree of muscle tone increased up to 6 years of age (p < 0.05), then decreased between 6 and 12 yrs of age (Figure 3).

At 4 years of age 47% of the total population of children with CP had spasticity in their gastro-soleus muscle graded as Ashworth 2–4. After 12 years of age 23% of the children had that level of spasticity.

**Figure 4:**
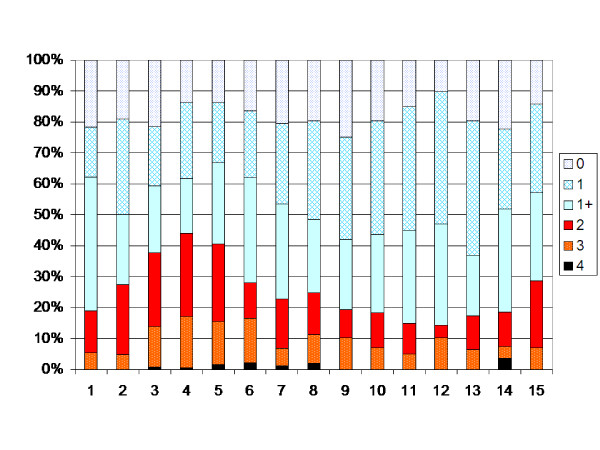
**Degree of spasticity in the gastrocnemius-soleus muscle according to the modified Ashworth scale related to age in the total sample of children with unilateral CP (186 children, 1269 measurements).** Number of measurements presented as a percentage of the total number of measurements in each age group.

**Figure 5:**
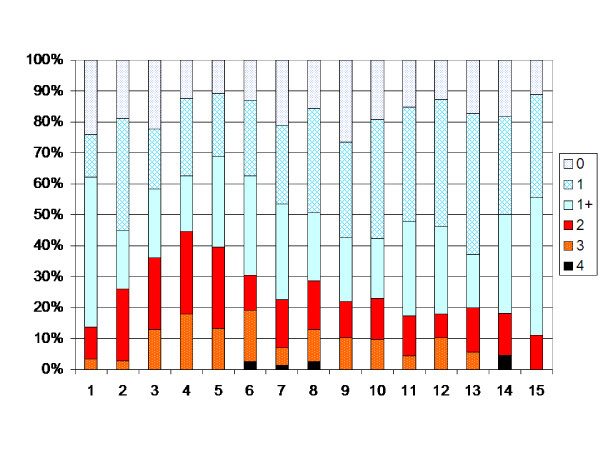
**Degree of spasticity children with unilateral CP according to Figure 4.** Children treated with TAL excluded.

The children with spastic unilateral CP showed an increasing muscle tone up to 4 years of age, followed by a decreasing tone up to 11 years of age (p < 0.05, Figure 4). After 11 years of age an increasing muscle tone was registered when children operated with TAL were included (p < 0.05). When those operated with TAL were excluded, no statistically significant change in muscle tone was seen after 7 years of age (Figure 5).

**Figure 6:**
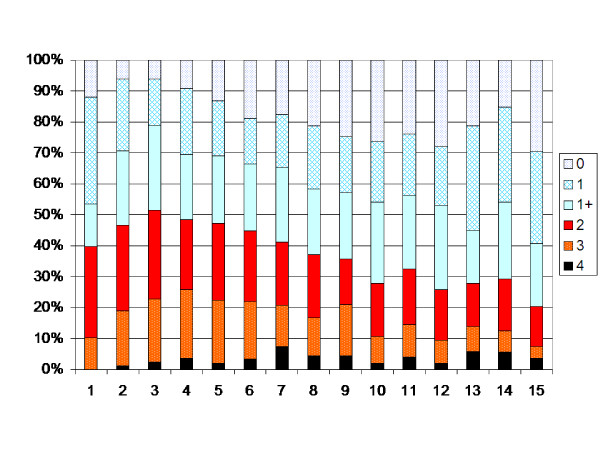
**Degree of spasticity in the gastrocnemius-soleus muscle according to the modified Ashworth scale related to age in the total sample of children with bilateral CP (266 children, 3521 measurements).** Number of measurements presented as a percentage of the total number of measurements in each age group.

**Figure 7:**
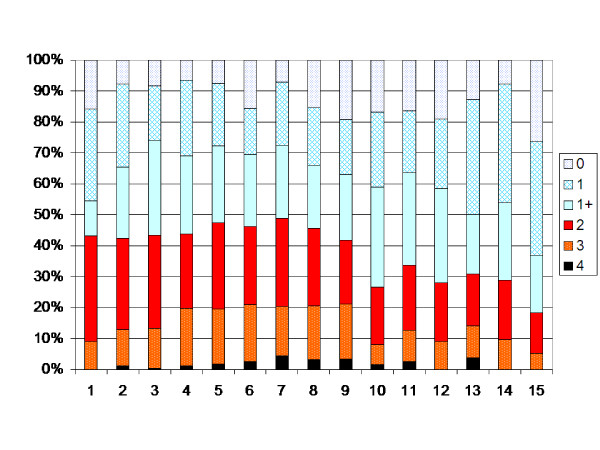
**Degree of spasticity in the children with bilateral CP according to Figure 5.** Children treated with SDR, ITB or TAL excluded.

The CP subtype spastic bilateral showed almost the same pattern as the total sample, with turning points at 3 and 12 years (Figure 6). Of the 266 children with bilateral CP, 66 had been operated with SDR, ITB or TAL (Table 4). Excluding these children, the turning points shifted to 7 and 12 years (Figure 7).

**Figure 8:**
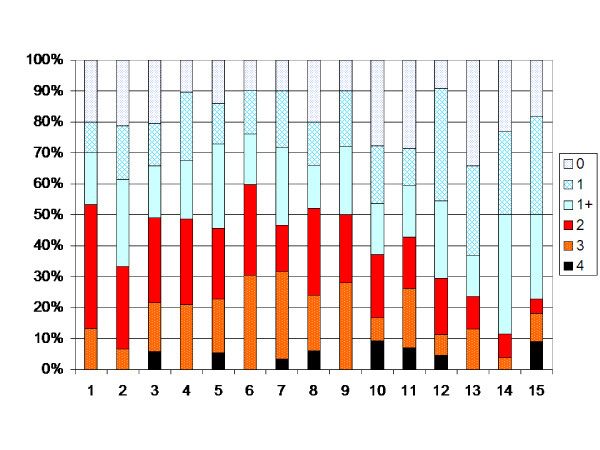
**Degree of spasticity in the gastrocnemius-soleus muscle according to the modified Ashworth scale related to age in the total sample of children with dyskinetic CP (72 children, 882 measurements).** Number of measurements presented as a percentage of the total number of measurements in each age group.

The children with the dyskinetic type of CP showed an increasing muscle tone up to age 6 (P < 0.05), followed by a decreasing pattern up to age 15 (p < 0.05) (Figure 8). Excluding the 9 children operated with ITB or TAL did not change the results.

**Figure 9:**
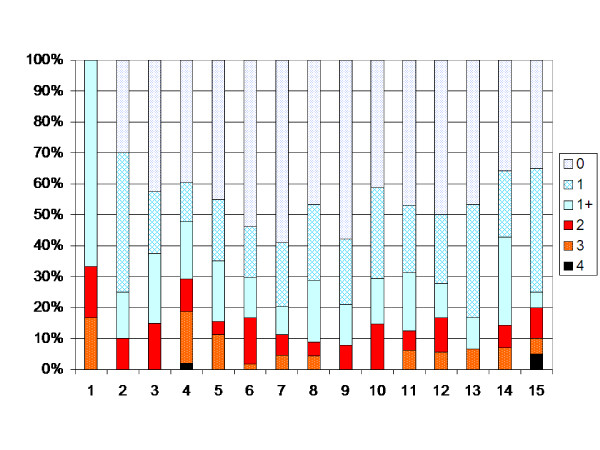
**Degree of spasticity in the gastrocnemius-soleus muscle according to the modified Ashworth scale related to age in the total sample of children with ataxic CP (53 children, 546 measurements).** Number of measurements presented as a percentage of the total number of measurements in each age group.

The children with ataxic type of CP had, as expected, on average a lower degree of spasticity (Figure 9). In the SCPE classification, children with ataxic diplegia are classified as ataxic CP. The number of measurements did not allow for analysis using Akaike's AIS.

## Discussion

The development of spasticity with age in children with CP has, to our knowledge, not been analysed before. The study showed an increasing muscle tone up to 4 years of age in children with spastic CP and up to 6 years in children with dyskinetic CP, followed by a decreasing tone up to 12 years of age.

In the 1990s two new methods to reduce spasticity in children with CP were introduced, ITB and BxA treatment. In the same period treatment with SDR was popularized [13]. These techniques made it important to quantify spasticity, both for analysing indication and for evaluation of treatment.

The modified Ashworth scale is the most widely used instrument for assessing spasticity, and it has been used in most outcome studies after treatment with SDR, ITB and BxA [1]. No other instrument is so simple that it can be used for screening purpose or clinical follow-up of larger samples. The modification from the original scale is the addition of one level of measurement (1+) at the lower end of the scale. There is no evidence supporting an ordinal relationship between level 1 and level 1+, and there is a lack of evidence for the relation between the introduced terms "catch" and "release" to the definition of spasticity as resistance to passive movement [14]. Some studies have shown problems with interobserver reliability with the Ashworth scale [15,16]. The reliability is better with the original scale, as is measuring plantar flexion of the ankle compared with measurement of more proximal muscles [15,17]. Some of these problems regarding scaling and reliability are reduced as the scale in the present study was dichotomized and grouping level 1 and 1+ together.

The main reason for dichotomizing the Ashworth scale was to make possible the modelling process in which we determine the number of age intervals and their corresponding endpoints. We wanted to estimate the development of spasticity with age with a method that would supply equivalent estimates and confidence intervals for all CP-subtypes. Additionally, the method should allow for the possibility of a change of magnitude and direction of this development. This was not likely to be possible with any other method.

It is sometimes difficult to distinguish limb stiffness resulting from muscle shortening from stiffness caused by spasticity [17]. As muscle stiffness increases with age [19,20] the problem with differentiating shortening from spasticity would result in an underestimation of the decreasing spasticity with age found in this study. It could also be an explanation for the increased muscle tone registered after 11 years of age in children with unilateral CP. The problem is reduced by grouping levels 2–4 together in the analysis. In the present analysis a low reliability would, in absence of systematic errors, only increase the observed variation in the population spasticity measurements, with increased confidence interval width. A low reliability should only affect our ability to correctly detect an actual change.

The validity of the Ashworth scale has been analysed in several studies [1]. Pandyan et al. [21] compared measurement of resistance to passive movement of the elbow with estimation of spasticity according to the modified Ashworth scale in adult stroke patients. They concluded that the modified Ashworth scale did not have sufficient validity to discriminate between the lover levels, but may be useful as a scale with fewer grades. Leonard et al. [22] compared the modified Ashworth scale with myometer measurement of the biceps brachii and found moderate to high correlation between the methods. Sköld et al. [23] compared EMG-measurement of the quadriceps with estimation of spasticity according to the modified Ashworth scale in adults with spinal cord injury. They found a positive correlation and concluded that the modified Ashworth scale accurately reflect movement-provoked spasticity. Each increasing grade on the Ashworth scale corresponded to increasing myoelectric activity levels.

Most of the children treated with BxA were 3–5 years of age at time of treatment (Figure 1). They were most often examined more than three months after treatment. As the majority of treatments were done at the same age period as the highest degree of muscle tone was found, compensation for a possible treatment effect would strengthen the pattern of increasing spasticity followed by decreasing spasticity with age.

Spasticity and decreased active muscle strength are the two predominant symptoms in children with CP [24]. The decreased active muscle strength is to some extent compensated by the passive strength that is created by spasticity. Thus, increased muscle tone is sometimes valuable, and treatment of spasticity should aim at modulating the muscle tone for their maximum benefit.

The findings in the present study may have several clinical implications. The decreasing spasticity with age is in accordance with the clinical findings that some children with toe walking gait, if they do not develop muscle shortening, after some years start walking with heel contact [25]. This could be a consequence of the increasing body weight and/or a result of decreasing muscle tone with age.

The decreasing spasticity with age must also be kept in mind when considering treatment with methods permanently reducing spasticity, such as SDR, or when considering TAL. Some children treated with SDR after some years, with increasing body weight and height, have increasing problems with muscle weakness [26]. Perhaps at that age they would have benefited from more passive muscle strength by a higher muscle tone. The finding that spasticity is not a constant phenomenon favours treatment with reversible methods, such as BxA and ITB. The decreasing muscle tone and the increasing weakness related to body weight and height must also be kept in mind when considering TAL. A crouch gait after TAL could be caused by over-lengthening, but also a consequence of a decreasing muscle tone of the gastrocnemius-soleus muscle.

The findings are also important for follow-up studies after spasticity reducing treatment. The decreasing muscle tone with age raises the need for control groups and long-term follow-up when evaluating treatments that permanently affect the muscle tone, muscle length and muscle strength, such as SDR and orthopaedic surgery.

## Conclusion

The present study showed that in children with CP, the muscle tone measured with the Ashworth scale increases up to 4 years of age and then decreases up to 12 years of age. The same tendency is seen in all spastic subtypes. This is, to our knowledge, the first report showing this development, and thus needs confirmation in further longitudinal studies. The findings may have implications both for clinical judgement and for research studies on spasticity treatment.

## Abbreviations

AIC: Akaike's An Information Criterion; BxA: Botulinum toxin A; CP: Cerebral Palsy; CPUP: Swedish follow-up programme for Cerebral Palsy; GMFCS: Gross Motor Function Classification System; ITB: Intrathecal Baclofen Pump; SCPE: Surveillance of Cerebral Palsy in Europe network; SDR: Selective Dorsal Rhizotomy; TAL: Tendo Achilles Lengthening.

## Competing interests

The authors declare that they have no competing interests.

## Authors' contributions

GH and PW designed the study. Both authors analysed the results. GH wrote the first draft, which was then actively improved and revised by both authors.

## Pre-publication history

The pre-publication history for this paper can be accessed here:


